# Multimodal microwheel swarms for targeting in three-dimensional networks

**DOI:** 10.1038/s41598-022-09177-x

**Published:** 2022-03-24

**Authors:** C. J. Zimmermann, P. S. Herson, K. B. Neeves, D. W. M. Marr

**Affiliations:** 1 Department of Chemical and Biological Engineering, Colorado School of Mines, Golden, CO USA; 2 Department of Anesthesiology, University of Colorado Denver, Anschutz Medical Campus, Aurora, CO USA; 3 Department of Bioengineering, University of Colorado Denver, Anschutz Medical Campus, Aurora, CO USA; 4 Department of Pediatrics, University of Colorado Denver, Anschutz Medical Campus, Aurora, CO USA

**Keywords:** Biomedical engineering, Magnetic devices

## Abstract

Microscale bots intended for targeted drug delivery must move through three-dimensional (3D) environments that include bifurcations, inclined surfaces, and curvature. In previous studies, we have shown that magnetically actuated colloidal microwheels (µwheels) reversibly assembled from superparamagnetic beads can translate rapidly and be readily directed. Here we show that, at high concentrations, µwheels assemble into swarms that, depending on applied magnetic field actuation patterns, can be designed to transport cargo, climb steep inclines, spread over large areas, or provide mechanical action. We test the ability of these multimodal swarms to navigate through complex, inclined microenvironments by characterizing the translation and dispersion of individual µwheels and swarms of µwheels on steeply inclined and flat surfaces. Swarms are then studied within branching 3D vascular models with multiple turns where good targeting efficiencies are achieved over centimeter length scales. With this approach, we present a readily reconfigurable swarm platform capable of navigating through 3D microenvironments.

## Introduction

Actively manipulated microbots present a promising platform for targeted delivery of therapeutic drugs^1,2^ by swimming through bulk fluid^3–6^ or by utilizing nearby surfaces to roll^7–10^ or walk^11^. Using applied magnetic fields, individual microbots, proposed for applications including microsurgery^12^, biofilm eradication^13^, blood clot removal^14^, and stem cell transplantation^15^ with structures incorporating helical^16^ or flexible components^17^, can travel against fluid flow^18,19^ or at speeds up to 600 µm/s^20^ in quiescent fluid. Though individual microbot translation can be accurately modeled^21^, applications involving therapeutic payloads will require significant microbot numbers and concentrations where swarming behaviors, such as those demonstrated in nature with insects, birds, and fish, have been observed. Such emergent structures include vortices^22–24^, ribbons^25^, carpets^26^, chains^27^, or dispersions^28^ composed of many individual microbots. In addition, swarms can be tuned to change modes to increase hyperthermia^29^, travel in confined spaces^22^, or increase translation in various bio-fluids^30^.

**Figure 1:**
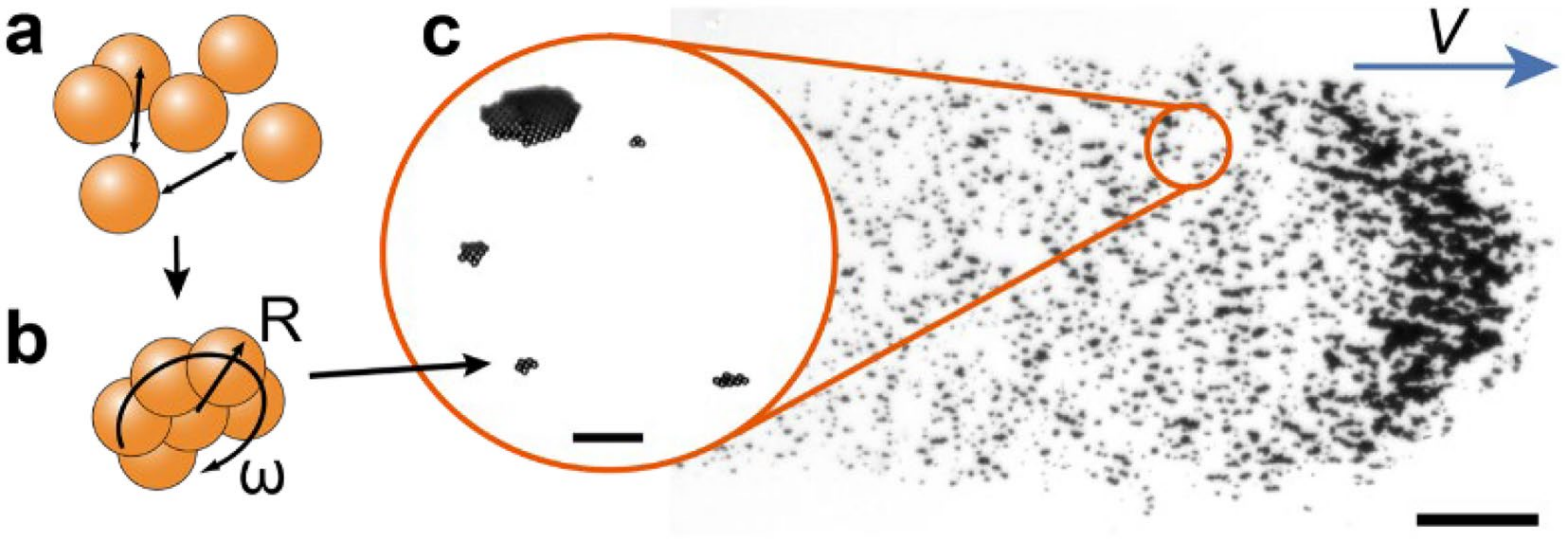
Upon application of a rotating magnetic field (a) individual 4.5 µm beads form into (b) µwheels which subsequently form into (c) swarms. Inset scale = 50 µm. Swarm scale = 300 µm.

While precise microstructures can be fabricated^31,32^ with good translational control^33^, microbots can be difficult to manufacture in bulk in the numbers required for therapeutic applications. Our previous work has focused on wheel-like microstructures (µwheels) that are reversibly and readily assembled in situ from superparamagnetic beads using a weak rotating magnetic field (Fig. 1). Before assembly, these individual particle building blocks are small enough to pass through the smallest capillaries in the body and, when assembled into µwheels, can translate at velocities over 200 µm/s^9^ on surfaces normal to gravity. For in vivo drug delivery however, µwheels will move as swarms (Fig. 1). Others have shown microbot swarms with multiple modes in 2D^22,27^, here the contribution is microbot swarm targeting in 3D. During treatment, µwheel swarms may traverse environments such as the circulatory, digestive, or urinary systems that are curved, not normal to gravity, and contain tortuous pathways. An effective platform must therefore be able to navigate highly-branching and inclined systems. To investigate these, we first characterize the behavior of component µwheels in 3D and develop strategies for swarm movement that enable faster translation, better climbing, wider spread, and mechanical action. Then, we investigate the targeting efficiency of µwheel swarms in a model 3D network inspired by the cerebrovasculature. Together, this work presents a complete approach for quickly assembling superparamagnetic beads in situ into concentrated yet highly efficient multimodal µwheel swarms that can adapt to their environment and target across centimeter length scales. With this, we present a microbot-based approach that is not limited to 2D environments and can effectively target within 3D vascular analogues.

## Results

### µWheel translation

**Figure 2:**
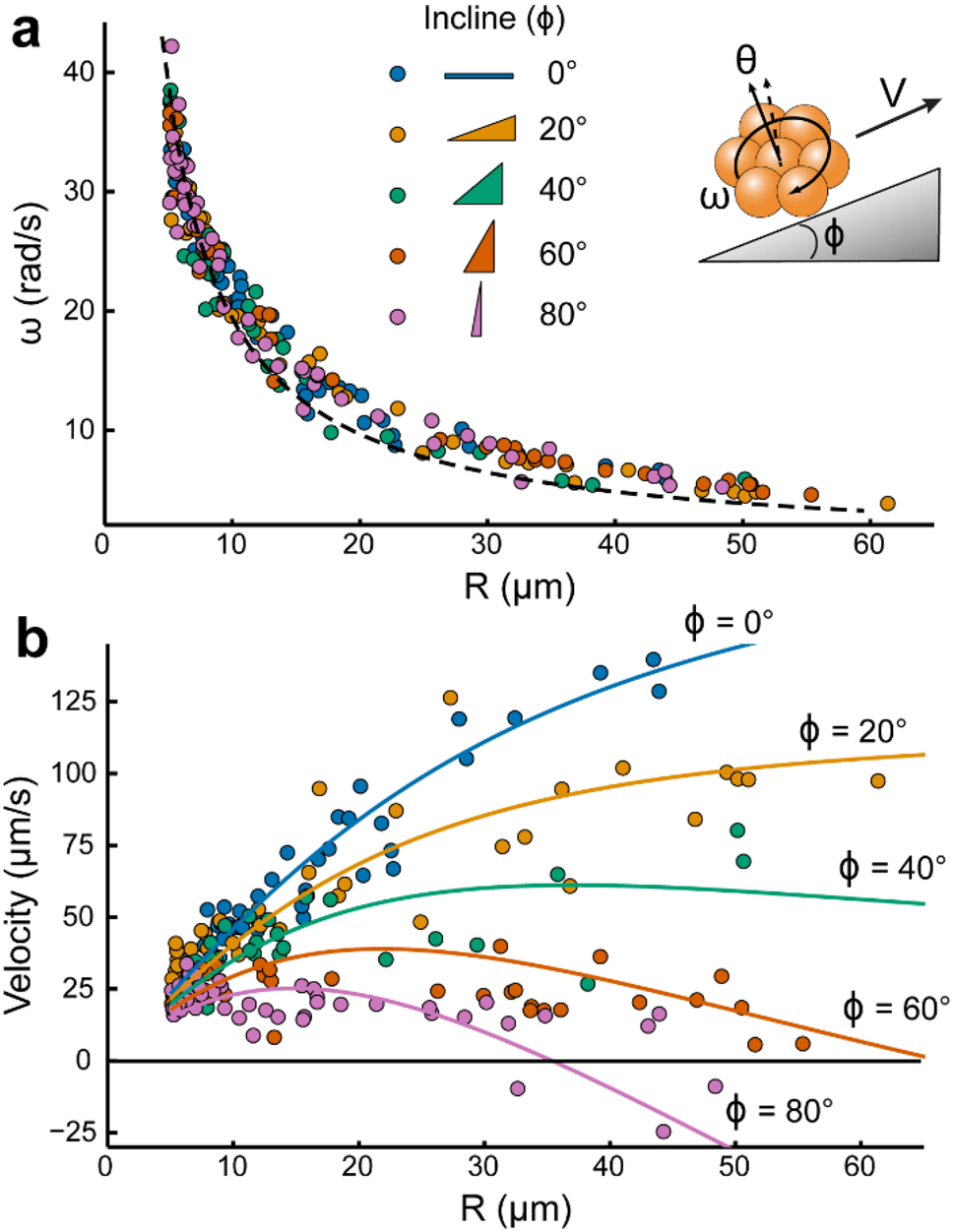
µWheel translation on inclines. (**a**) µWheel angular velocity ($\omega$) as a function of size ($R$) and incline angle ($\upphi$). Dotted line shows the $\omega \propto 1/R$ scaling. (inset) Translating µwheel on an incline. (**b**) µWheel velocity over incline angles 0–80° with solid lines the variable gap width model (Supplementary Equation 1). All µwheels were propelled with a constant 40 Hz circular rotating field of magnitude 3.7 mT and 30° camber angle (θ).

Essential for predicting movement in realistic geometries, we begin by describing the behavior of individual µwheels on inclined surfaces where, upon application of a rotating weak magnetic field (~ 4 mT), µwheels assemble from 4.5 µm Dynabeads^®^ into spinning clusters. While other superparamagnetic beads could be used, these highly-monodisperse particles consist of iron oxide domains within a polystyrene matrix, a relatively biocompatible material available at sizes that can be readily phagocytosed upon µwheel disassembly^34,35^. In addition, their surfaces can be easily functionalized to create drug delivery vehicles as previously demonstrated^14^. When oriented with a component normal to the surface, µwheels roll at velocities which depend not only on the µwheel rotation rate, but also on the size (Fig. 2a) and the camber, or tilt, angle θ of the µwheel relative to the surface normal. For this study we hold θ constant, focusing on the size and incline dependence of µwheel velocity. Unlike macroscopic wheels which move by gripping a solid surface, µwheels roll on an intervening layer of fluid and use wet friction to move. Their translational velocity can be predicted by balancing translational fluid drag and wet friction with the surface^9^. However, for translation up inclined surfaces the normal force, the µwheel distance from the surface, and the resulting frictional force are all altered. Accounting for this variable gap width, we develop a model (Fig. 2b and “Materials and methods”) that is valid across a broad range of incline angles 0–80°, enabling µwheel translation predictions while targeting.

With rotating magnetic fields of magnitude *H*, the induced magnetic torque can be expressed as^36^ $\tau =n\nu {\mu }_{o}{\chi }^{{{\prime}}{{\prime}}}{H}^{2}$ where *n* is the number of beads in the µwheel, $\nu$ is the volume of an individual bead, ${\mu }_{o}$ is the permittivity of free space, and ${\chi }^{{{\prime}}{{\prime}}}$ is the imaginary part of the magnetic susceptibility. By approximating the viscous rotational µwheel torque with that of a disk^37^ $\tau =32\eta \omega {R}^{3}/3$ and equating torques, one expects a µwheel rotational frequency of $\omega =3n\nu {\mu }_{o}{\chi }^{{{\prime}}{{\prime}}}{H}^{2}/32\eta {R}^{3}$. With a µwheel radius $R\sim {n}^{1/2}$ and for constant magnetic field strength we expect $\omega \sim 1/R$, a behavior we observe at all incline angles (Fig. 2a), suggesting that the rotational drag dominates over drag from the surface.

As expected, small µwheels with *R* < 10 µm are less affected by a change in incline due to their lower weight (Fig. 2b). However, large µwheels (> 35 µm radius), despite having significantly greater velocities on flat surfaces, are hindered by their mass and decrease in velocity. As a result and as the incline angle increases to 80°, µwheels greater than 35 µm in radius begin to slip backwards downhill as they roll (Supplementary Video 1). We also note that small µwheels (~ 10 µm radius) show only a 50% decrease in velocity at an 80° incline angle compared to 130% with large µwheels (~ 60 µm radius), suggesting that only a small load force (< 1 pN) is sufficient to retain proximity to the surface and translate up steep slopes.

### µWheel swarming

For applications requiring therapeutic payloads for example, large numbers of µwheels will exhibit collective swarming behavior or modes which can be switched depending on the field and the subsequent component µwheel motion. At these high concentrations, µwheels collide and combine to form larger µwheels; however, we observe steady-state µwheel size distributions where growth plateaus. For example, for a 3.7 mT rotating magnetic field, the maximum observed µwheel size is *R* ~ 60 µm. To understand this, we employ the Mason number^38^, relating viscous to magnetic forces, Mn = Pe/λ with Pe the Peclet number, relating convection to diffusion, and λ the dimensionless dipole strength^39^ given by $\lambda ={\pi \mu }_{0}{a}^{3}{\chi }^{2}{H}^{2}/9kT$. By considering an individual bead at a µwheel edge we have ${\text{Pe}}=6\pi \eta {a}^{3}\dot{\gamma }/kT$ and ${\text{Mn}}= 54\eta \dot{\gamma }/{\mu }_{0}{\chi }^{2}{H}^{2}$ with $\dot{\gamma }$ the shear rate. With Mn ~ $\dot{\gamma }$, small $\dot{\gamma }$ lead to small Mn where magnetic interactions create µwheel stability; however, as $\dot{\gamma }$ and Mn increase, viscous forces dominate, providing a significant energetic barrier to uncontrolled agglomeration in high shear environments. Probing further in the context of a rotating µwheel, we approximate the shear rate $\dot{\gamma }$ at the outer edge with $\omega R/l$ where $l$ is a characteristic fluid velocity decay length to obtain ${\text{Mn}}= 54\eta \omega R/{l\mu }_{0}{\chi }^{2}{H}^{2}$ or Mn ~ $\omega R$. Now recognizing from Fig. 2a that, for constant fluid and field conditions, $\omega \sim 1/R$ and little variation in Mn with µwheel size is expected in our experiments. As a result, and due primarily to this slowing rotation with larger µwheels, we would expect continued growth if sufficient beads are available without changes in the applied field.

**Figure 3:**
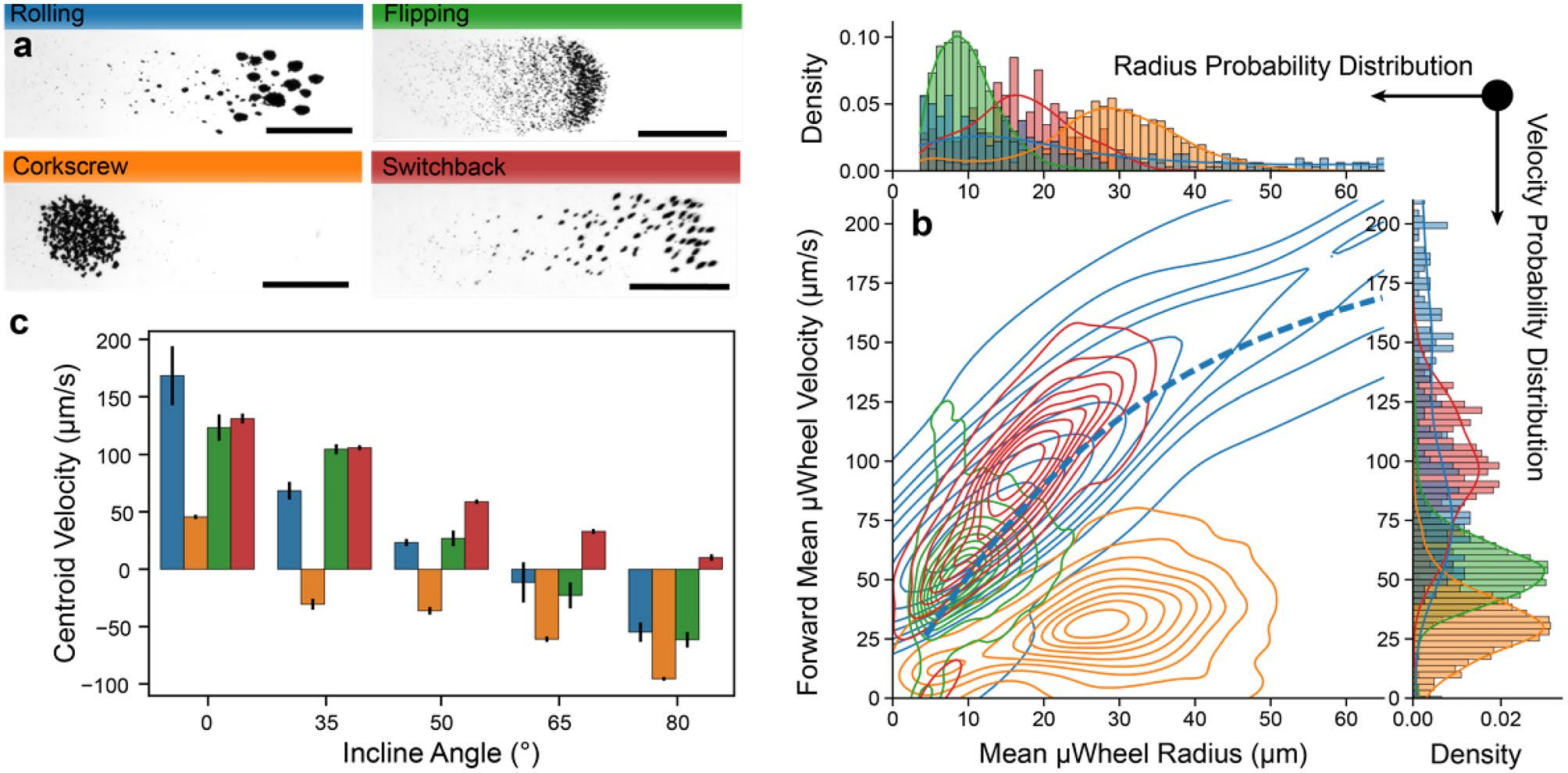
µWheel swarm characterization. (**a**) Optical microscopy of four swarm modes after ~ 15 s of magnetic actuation. Scale = 1 mm. (**b**) “Fingerprint” of each swarm mode. Blue dashed line is the model line at 0° from Fig. 2b. Each probability distribution is fit with a kernel density estimate to guide the eye. (**c**) Centroid, or center of mass, velocity of swarms at 0° and multiple steep angles. Error bars represent standard deviation of triplicate videos.

An effective approach for introducing shear and disrupting µwheel growth is with sudden changes in the heading direction or camber angle of the rotating field, where µwheels can separate into smaller components. Using different patterns of heading directions and camber angles (Supplementary Fig. 1) and without varying the field strength or frequency, µwheel size distributions can be created and specific swarm modes specializing in various tasks designed. Here, we report four unique µwheel swarm modes which correspond to specific needs when targeting (Fig. 3a): rolling mode, for optimal mass flux with quickly moving µwheels; switchback mode, for steep incline traversal using rapid turns; flipping mode, for deposition of small µwheels across a large area where µwheels are forcibly broken apart; and corkscrew mode, to support mechanical action by translating forward with a helical motion for enhanced penetration^14^ (Supplementary Video 2).

Rolling mode is the simplest and results when the applied field is unchanged with the heading and camber angles kept constant (Supplementary Fig. 1). Specializing in high mass transport, rolling mode forms the largest µwheels that can quickly move across macroscale distances. As expected, it has the highest centroid velocity of studied swarms due to large µwheels; however, due to the lack of control on the µwheel size, a large range is formed, resulting in a significant spread. Flipping mode imparts rapid changes in camber angle which break up large µwheels resulting in small component µwheels. These smaller µwheels fall behind a concentrated front that breaks up with every change in camber angle, resulting in a deposition effect where beads are spread out over larger areas. Corkscrew mode, which has shown superior penetration into fibrin gels^14^, uses a helical motion and gradually changing camber angles. This swarm sacrifices translation speed but retains a mid-range size distribution. The changing heading and camber angle (Supplementary Fig. 1) enables this swarm to find the path of least resistance and avoid the formation of the largest µwheels. The helical motion due to the change in heading angle results in the slowest swarm mode. Switchback mode retains a constant camber angle but rapidly changes the heading direction, resulting in medium sized µwheels which perform better on inclines.

We track µwheel swarm movement at both the constituent µwheel (Fig. 3b) and bulk (Fig. 3c) levels and note that, for all swarm modes, the magnitude and frequency of the rotating magnetic field is kept constant. Using particle tracking, we obtain a unique “fingerprint” (Fig. 3b) that describes the influence of field actuation on the component µwheel radii and velocity. For example, flipping mode selects small µwheels from 4.5 to 20 µm radius while rolling has a much broader size distribution. Likewise, the velocity probability distributions show that the back-and-forth helical motion of the corkscrew mode exhibits the lowest forward velocity. In general, the velocity and radius distributions are related; the larger the component µwheels, the faster the swarm translates. For comparison, we include the data of Fig. 1b for individual µwheels along with the rolling swarm (Fig. 2b, dotted blue line) where the swarm-induced broadening of the velocity distribution is apparent, suggesting that, while presumably weak, µwheel-µwheel interactions are having a significant impact on overall behavior.

To determine swarm mode climbing ability, we measure swarm bulk velocity up steep inclines (Fig. 3c). In this, gravity slows swarm movement as angle increases with the switchback mode the only one able to translate up nearly vertical inclines. Such an alternating heading direction lowers the effective incline angle where the switchback angle φ, here 35°, results in a lower effective climbing angle ${\upphi }_{e\mathrm{ff}}={\mathrm{sin}}^{-1}(\mathrm{sin}(\upphi )\mathrm{cos}(\mathrm{\varphi }))$. For example, at $\upphi =80^\circ$ choosing a switchback angle of φ = 35˚ results in ${\upphi }_{\mathrm{eff}}\approx 54^\circ$. This technique is advantageous as it allows surface-enabled µwheel swarms to continue to move effectively up very steep angles without the use of additional external fields or magnetic field gradients. As ϕ increases, effective load decreases and µwheels, which require a load force normal to the surface and wet friction to translate, can no longer break symmetry and translate and simply sink due to their high density (Supplementary Video 3).

### Targeting µwheel swarms in a three-dimensional network

**Figure 4:**
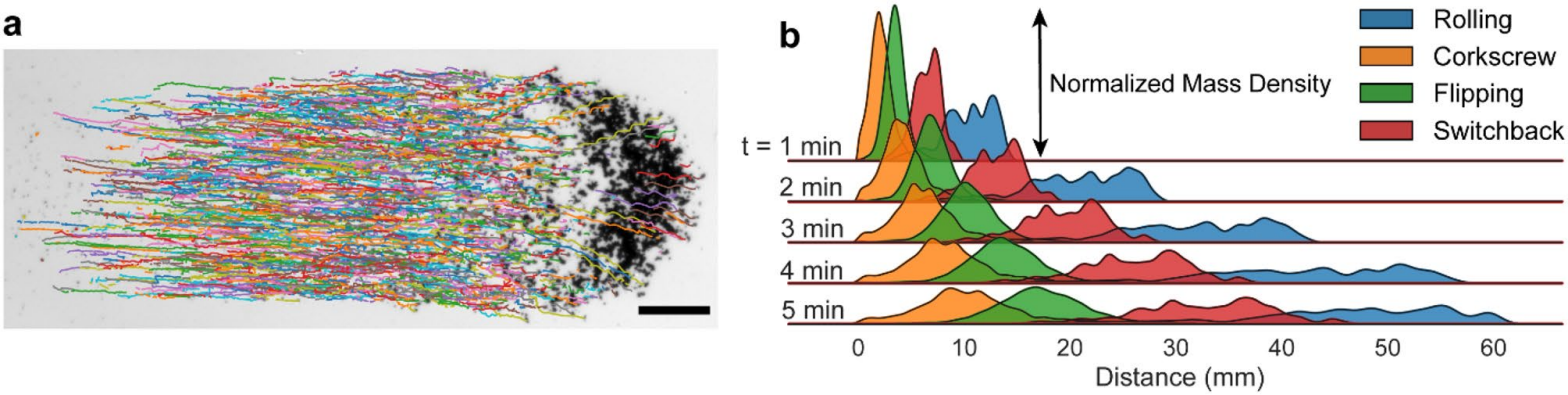
Spreading of µwheel swarms. (**a**) Sample trajectory tracking in a flipping swarm after 20 s. Scale = 300 µm. (**b**) Predicted mass transport of swarms based on measured velocity distributions. The shaded area under each curve is equal.

An effective drug delivery system requires sufficient microbot mass to reach the target area to provide a therapeutic drug dose. As all swarms are assembled and controlled using a global field, individual component control is not possible and variation in µwheel velocities causes a swarm mode dependent spread. Using tracking data from the swarm study (Fig. 4a) and assuming a constant µwheel distribution, a quantitative time-dependent mass distribution can be readily predicted (Fig. 4b). Targeting with a global field requires the heading angle of the swarm to be changed when the most mass is near a target turn. While switchback mode exhibits decreased spread over long distances, rolling swarms provide the fastest mass transport of the swarm modes investigated here (Fig. 3c). We therefore use rolling mode to quickly move mass over significant distances (~ 10 mm/min) for our study of payload targeting in macroscale 3D networks.

**Figure 5:**
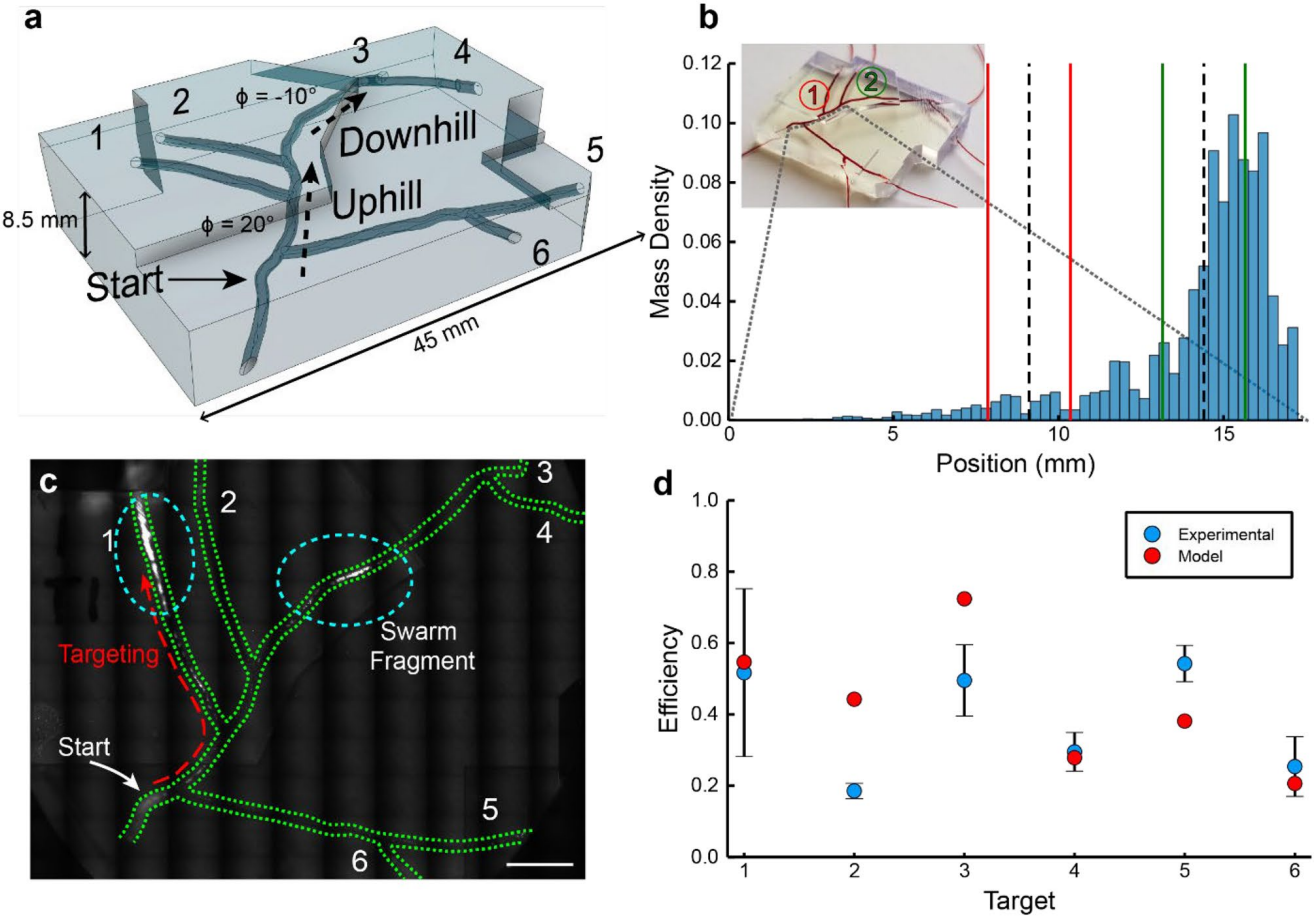
µWheel swarm targeting. (**a**) CAD model of branching 3D vascular model. Each branch is labeled 1–6. Image created using Fusion 360 (Autodesk). (**b**) µWheel mass density histogram of rolling swarm simulated ~ 14 mm into the device. Dotted lines signify the locations of the center of branches 1 and 2, while the bordering solid lines select which µwheels are captured in each branch. (**c**) Fluorescence scan after targeting µwheel swarm to branch 1, annotated with Illustrator (Adobe). Scale = 5 mm. (**d**) Experimental and modelled targeting efficiency of rolling µwheel swarm. (**b**,**d**) created using Julia v1.6.1 (https://doi.org/10.1137/141000671), annotated with Illustrator (Adobe).

As a platform to test targeting, we fabricated clear 3D-printed branching models from an available murine cerebral model^40^ and vascular atlas^41^ of the murine middle cerebral artery (Fig. 5a). In this device, we conducted 18 independent experiments targeting each of the six branches in triplicate to determine the µwheel swarm targeting efficiency, defined as the mass of beads in the target divided by the total mass injected (Fig. 5d). For this study, the swarm was optically tracked and directed in real time. Using fluorescently-tagged µwheels, targeting efficiency can be directly observed after actuation (Fig. 5c). From these experiments, we demonstrate that µwheel swarms can be translated over centimeters up and down inclined cylindrical channels with diameters ~ 0.6 mm and directed to a target vascular branch with efficiencies averaging ~ 38%. As expected, the targeting efficiency generally decreases as the distance from the start and the number of intermediary turns increases.

To predict targeting efficiency in a vascular mimic, we model µwheel swarm translation with an injection mass whose distribution evolves in time (Fig. 4b). This allows a prediction of the evolution of the µwheel swarm at any point during targeting. In this simplified approach, we first use the previously measured component µwheel velocity and size distributions ($V\left(R, \phi \right),$ Fig. 2a) to simulate the makeup of the swarm which can be integrated forward in time. Next, we recognize that a turn results in a fragment of the swarm tails being lost to untargeted branches (Fig. 5b). Finally, the position of each µwheel ${x}_{i}$ in the swarm is calculated through

1 $$\begin{array}{c}{x}_{i}={\int }_{{t}_{start}}^{{t}_{end}}V\left({R}_{i}, {\phi }_{i}({x}_{i})\right)dt\end{array}$$

with ${\upphi }_{i}$ the incline angle evaluated for each time step $dt$ at the position ${x}_{i}$. This is achieved by encoding the 3D geometry of a network into a function ${\upphi }_{\mathrm{i}}\left({x}_{i}\right)$. Despite the simplicity of the model, we can predict targeting efficiencies within ~ 25% of the measured values (Fig. 5d) using simple integration and histogram clipping, demonstrating the flexibility of this technique.

## Discussion

We demonstrate here a potential drug delivery platform created from surface-enabled microbot swarms with switchable behavior, including targeting, penetration, climbing, and spreading. Swarms and the µwheels that comprise them are readily assembled in any desired quantity from commercially available superparamagnetic beads. Since the constituent beads do not attract without an applied external magnetic field, they reversibly disassemble into components small enough to pass throughout the circulatory system when the driving field is removed and transitions between swarm modes can be rapidly implemented. While surfactant was used here to decrease sticking, the beads could instead be functionalized with PEG^42^ also increasing biocompatibility^43^. Our approach uses only weak magnetic fields for both assembly and movement and has been demonstrated here in low flow environments such as those associated with the vasculature near occluded channels^44^. We note that this approach is directly compatible with higher strength and other externally applied fields^19,45^. Such a combination of fields may enable future swarm-scale targeting in flowing vascular networks, whole blood, or angles > 80° using an additional load force applied normal to the surface^45^. For flow, swarm modes with smaller component µwheel radii, such as the flipping or switchback swarms, would be best for sampling where fluid velocity is lowest.

Because µwheels are reversibly assembled from individual beads with application of a magnetic field, the effects of field switching are immediate (~ 10 ms) and achieved at the sub-µwheel level. As a result, µwheel swarms can readily switch modes, allowing adaptable travel through complex environments. Rolling mode with large µwheels could be used for quick translation until a steep incline is reached where switchback mode would be used. Flipping mode forms the smallest µwheels and could then be used to navigate in the most constrained spaces. After reaching the target site, flipping mode could be used to spread µwheels across a target patch (Supplemental Video 4) or corkscrew mode could be used to penetrate through a blockage. Our work to date has demonstrated the use of corkscrew mode on fibrin gels^14^ where the helical turn and camber angle rotation frequencies could be readily tuned depending on the target blockage. Of significant note here is that all assembly, translation, and swarm movement for all modes do not require gradient magnetic fields which are difficult to scale to larger sizes but instead use a global weak field (~ 4 mT) that acts uniformly through the working volume.

While we observe that µwheel swarms have weak µwheel-µwheel interactions that lead to swarm dispersion, over macroscale distances they can still achieve good targeting efficiencies (Fig. 5d) which are not limited by swarm velocity, but rather by swarm dispersion and global control. While other researchers have successfully tuned paramagnetic swarming microbots for attractive interactions^27^, they often report low (< 10 µm/s) swarm velocities. Our approach emphasizes targeting speed as an important variable, especially important for treatment of diseases like stroke where timing is critical. If translation with lower spread is needed, other modes can provide a more cohesive swarm due to their lower µwheel size variance (Fig. 4b).

We show that µwheels can translate up steep inclines and their behavior is captured with a variable gap width model. We calculated these gaps across a wide range of incline angle and µwheel radii (Supplementary Fig. 2) by a simple balance of load and electrostatic repulsion and confirmed that gap width decreases up to 70% with increasing load force. Surprisingly, µwheels can travel up inclines as steep as 80˚, providing access through most geometries and showing that swimming microbots are not required to locomote in complex 3D networks. They do so by using switchback turns and selected optimal µwheel sizes to transport large quantities of beads up steep inclines, an actuation approach that greatly extends the 3D locomotion ability of surface-enabled microbot swarms.

Finally, we demonstrate that µwheel swarms can quickly and successfully target in 3D environments. By combining our individual µwheel model with swarm component radius distributions, we can predict whole swarm movement and targeting efficiency within ~ 25% through arbitrary 3D geometries. While we currently direct µwheel swarms manually via joystick for all presented experiments, future work could automate turn heading angle changes for autonomous control if real-time tracking is difficult in the target geometry. Additionally, high resolution scans of target systems in the body are routinely conducted for a priori knowledge of the targeting environment. Not only is this important to identify treatment location, but the exact layout can vary widely from person to person^46^. This emphasizes the value of techniques that can predict microbot distributions and targeting efficiencies in arbitrary targeting pathways with inclines and bifurcations. We focused here on rolling mode due to its superior mass transport and lack of steep inclines in the target model. Future work will focus on optimization strategies for switching between modes depending on the challenges of a particular target network.

## Materials and methods

### Rotating microscope

**Figure 6:**
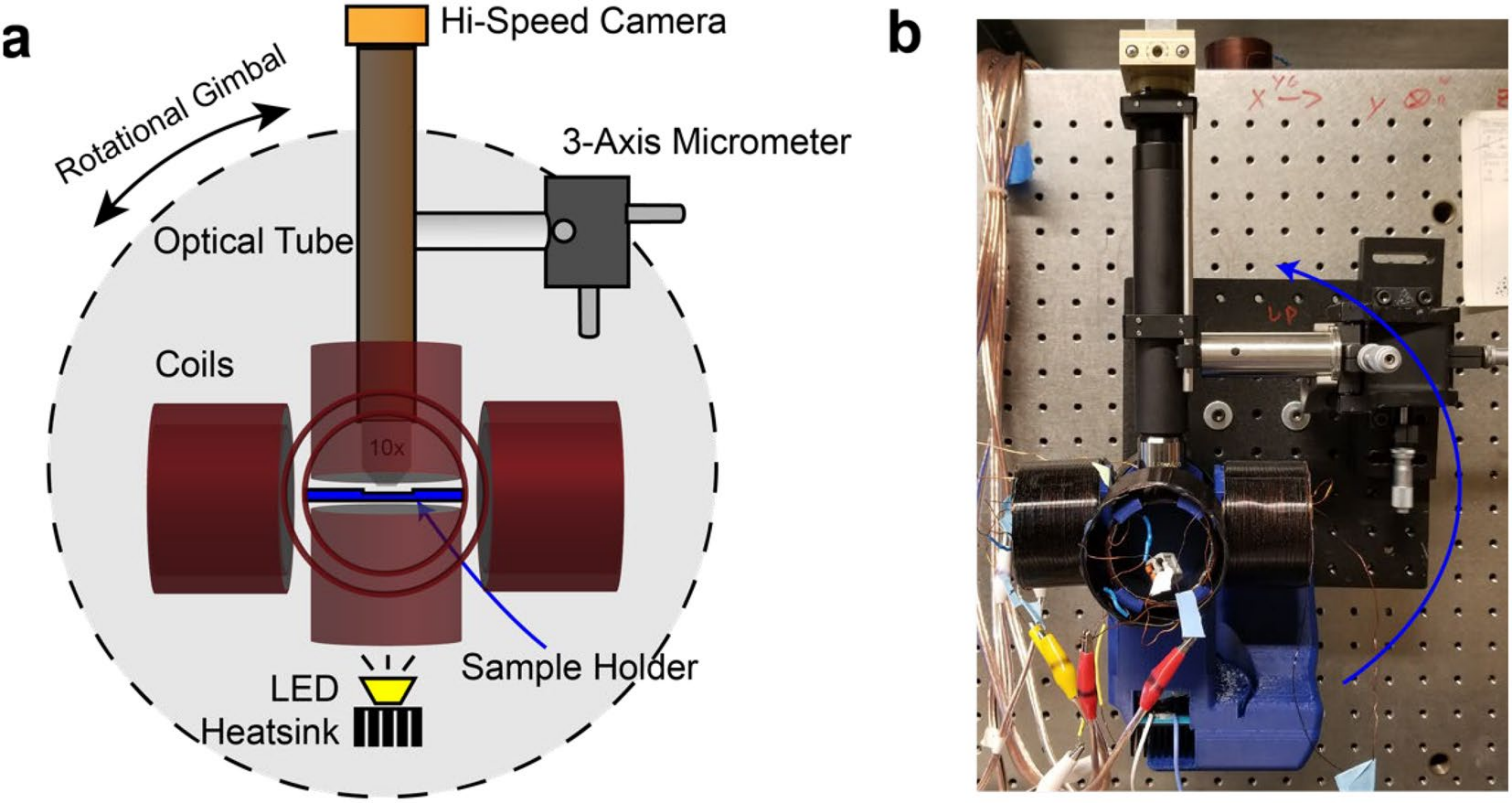
Rotating microscope and actuation apparatus, created and annotated using Illustrator (Adobe). (**a**) Diagram depicting the optical train and coils. 10 coils in total are depicted here, with 2 sets of concentric coils on the *x* and *y* axes and 2 individual coils on the *z* axis. (**b**) Fabricated apparatus. The body is 3D-printed out of poly-lactic acid (PLA) polymer while the gimbal is mounted behind the black optical board.

Visualization of µwheel translation on non-horizontal surfaces requires an apparatus in which the incline angle can be changed while keeping the electromagnet and microscope arrangement fixed. To achieve this, a custom-built 3D printed microscope and actuation system was fabricated (Fig. 6) where coils supply the rotating magnetic fields in tandem with established signal generation software^47^. The z axis consists of two 50 mm i.d. 400 turn coils above and below the sample. The x and y axes have two pairs of coils each of which are comprised of a 50 mm i.d. 400 turn coil and 65 mm i.d. 400 turn outer coil. The optical train consists of a high-speed camera (Epix SV 643 M), optical tube, and switchable objective (Olympus) attached to a three-axis micrometer for movement of the optical train along with a dimmable LED light source (Luxeon 5000 K, Alberta, Canada). The entire device is attached to a precision rotation stage (UTR120, Newport) to allow for specification of incline angle.

### Component µwheels on inclines

The sample chamber consists of two square 22 mm glass cover slips of 0.17 mm thickness sandwiching a 0.5 cm ID gasket cut from double-sided tape (RP32 VHB™ tape, 3 M, Maple, MN). This 800 µm tall gasket allows for large µwheels to travel unimpeded by the top surface. A 12 µL sample of 4.5 µm superparamagnetic beads (Dynabeads^®^ M-450 Epoxy, Thermo Fisher) diluted 125× with 0.2 weight % sodium dodecyl sulfate (SDS) (Sigma-Aldrich) is used for all µwheel experiments to prevent aggregation between particles and surfaces. Additional 0.2% SDS solution was added to fill up each sample chamber before closing. A standard rotating magnetic field was used for all experiments with a field strength of 3.7 mT, 40 Hz, and 30° camber angle^47^. All µwheel tracking was performed using custom particle tracking software MuTracker with the complete source available on GitHub^48–51^. The rotation rate of most µwheels was calculated through the fast Fourier transform of the rotation angle of the fit ellipse. The highest peak of the frequency domain corresponds to one half of a µwheel rotation. This method fails for radially symmetric µwheels and, in this case, the rotation rate was determined manually.

We describe motion on inclines using a model where the µwheel weight, $W$, can be expressed as $W=mgn$ with $N$ the normal force, $m$ the mass of an individual bead, $g$ the gravitational acceleration, and $n$ the number of beads in a µwheel. Though previous work has considered a camber angle dependent load^9^, here we omit because the camber angle used is small and held constant. For inclines in 3D, we split the weight into a normal $y$ component, ${W}_{y}=mgn \mathrm{cos}(\upphi )$, and a parallel $x$ component, ${W}_{x}=mgn \mathrm{sin}(\upphi )$, with $\upphi$ the incline angle (Fig. 2a inset). We have previously shown that the fluid gap, $\updelta$, between a µwheel and the surface is analogous to the hydrodynamic lubrication region that exists between two fluid-lubricated solids^45^. The load force varies widely due to $R$ and $\upphi$ suggesting an approach that considers a variable fluid gap width. To approximate this gap width for a wide range of µwheel radii (5–70 µm) and incline angle (0–80°), we balance the forces in the normal $y$ direction:

2 $$\begin{array}{c}\sum {F}_{y}={{W}_{y}-F}_{es}=0\end{array}$$

with ${F}_{es}$ the electrostatic force, approximated for an individual bead wall interaction by^10,52^:

3 $$\begin{array}{c}{F}_{es}=4\pi \epsilon {\epsilon }_{0}a\kappa {\nu }_{1}{\nu }_{2}{e}^{-\kappa \delta }\end{array}$$

with $\epsilon$ the permittivity of the medium, ${\epsilon }_{0}$ the vacuum permittivity, $a$ the radius of a bead, ${\kappa }^{-1}$ the Debye length, and ${\nu }_{1}$ and ${\nu }_{2}$ the zeta potential of the particles and the near surface, respectively. However, because of asymmetry and spin, the actual distance between the wall and µwheel will not be that of a simple sphere. To account for this we include a fitting parameter ${k}_{1}$ and solve for the gap width to obtain:

4 $$\begin{array}{c}\delta ={k}_{1}{\kappa }^{-1}\mathrm{ln}\left(\frac{4\pi \epsilon {\epsilon }_{0}a\kappa {\nu }_{1}{\nu }_{2}}{mgn\mathrm{cos}\left(\phi \right)}\right) .\end{array}$$

With experimental data we find ${k}_{1}$ = 1.50, indicating the observed non-circularity of measured µwheels across the large range of radii. Calculated separation heights are in Supplementary Fig. 2. We next balance the forces in the direction of propulsion $x$:

5 $$\begin{array}{c}\sum {F}_{x}={F}_{f}-{F}_{d}-{W}_{x}=0\end{array}$$

with ${F}_{f}={\mu }_{k}{W}_{y}$ the wet friction force and ${F}_{d}$ the drag force. We approximate ${F}_{d}$ using an analytical solution for viscous drag on a cylinder^53^

6 $$\begin{array}{c}{F}_{d}={k}_{2}\frac{{8}\pi a\eta V}{\mathit{ln}\left(\frac{2\left(R+\delta \right)}{R}\right)-\frac{1}{4}{\left(\frac{R}{R+\delta }\right)}^{2}}\end{array}$$

Along with another fitting parameter ${k}_{2}$ for irregular µwheel shape with $\eta$ the dynamic viscosity and $V$ the velocity of the µwheel. With experimental data we determine that ${k}_{2}=3.11$ suggesting that the increased surface area caused by a collection of spheres compared to a smooth cylinder systematically increases the drag force. To determine the wet friction coefficient ${\mu }_{k}$, we balance the frictional torque, ${T}_{f}={\mu }_{k}{W}_{y} \times R$, with the torque required to shear the lubrication fluid layer, ${T}_{shear}=\tau A \times R$, with $A$ the contact area and the fluid shear stress $\tau$, which by assuming a linear velocity profile can be expressed as^9^

7 $$\begin{array}{c}\tau =\eta \frac{\partial V}{\partial y} \approx \eta \frac{\omega R-V}{\delta }\end{array}$$

By then approximating the contact area of the µwheel $A\approx Ra$, one obtains

8 $$\begin{array}{c}{\mu }_{k}=\frac{\eta a}{\delta {W}_{z}}R\left(\omega R-V\right)\end{array}$$

with $\omega$ the angular velocity. Finally, using $n\approx {\left(\frac{R}{a}\right)}^{2}$ we arrive at a model that can predict the velocity of component µwheels across wide ranges of incline angles and radii (Eq. S1, Fig. 2b). Supplementary Equation 1 with fit parameters ${k}_{1}$ and ${k}_{2}$ is used to predict individual µwheel velocity during targeting in 3D models.

### µWheel swarms

An identical sample chamber, solution, and magnetic field (see µWheels on Inclines) was used for all swarm experiments, except for an increased bead solution of 15 µL. Before each video, a permanent magnet was used to collect beads on the top of the sample chamber before removing the magnet and letting the beads settle for ~ 10 s. Each swarm mode video was performed in triplicate across all incline angles presented here (see “Rotating microscope”).

Particle tracking was performed using open source software MuTracker^51^ which identifies and tracks the trajectories of all µwheels. The output of the software is the mean µwheel velocity and the mean radius of every µwheel in the swarm. However, in the event of a collision between two µwheels, one trajectory ends while the other’s velocity and radius is increased to the mean before and after the collision. The reverse occurs during a splitting event.

### 3D printed mouse middle cerebral artery model

We fabricate 3D microfluidic devices using a Form 3 stereolithography (SLA) 3D printer (Formlabs Inc, Somerville, MA). Using available models of the mouse brain from the Allen Reference Atlas^40^ and a mouse cerebrovascular atlas^41^, a tracing of the MCA was projected onto the curvature of the surface of the brain using computer-aided design software (Fusion 360, Autodesk). A selection of the MCA was cropped and designed into an enclosed microfluidic device with seven outlets and multiple viewing windows. Each inlet was plumbed with 0.01″ ID by 0.030″ OD clear Tygon^®^ tubing (Cole Parmer) and fixed in place with two-part epoxy. A new microfluidic device was printed for each experiment to avoid residual fluorescence from stuck particles at junctions.

### Fluorescently labeled beads

To enable targeting efficiency quantification, 20 µL of 4.5 µm epoxy Dynabeads^®^ were incubated in 0.1 mg/mL rhodamine B (Sigma Aldrich) for 24 h. After incubation, the beads were washed five times with 0.2 wt% SDS solution. The bead solution was stored in darkness until used, and, if greater than 5 h elapsed, were washed 3× using the same process to remove any desorbed rhodamine B.

### Targeting in MCA model

To demonstrate targeting, the fabricated MCA microfluidic devices were first loaded with 0.2 wt % SDS in deionized water. Fluorescently labeled beads were localized at a defined start area using a permanent magnet while 20 µL of fluorescently labeled bead solution was injected into the closest bottom inlet. All tubing was clamped, and the device placed on a fluorescence-capable microscope with a motorized stage (Olympus IX81). A full scan using a TRITC filter was performed before each experiment to determine the number of beads injected. The device was then placed for observation and targeting on a separate fluorescence microscope (Olympus OpenStand) fitted with five identical 50 mm i.d. 400 turn coils. These coils are similar to those used for the rotating microscope except they lack a sixth coil above the sample. Using a joystick to orient the 3.7 mT rotating field, the particles were assembled into a swarm and directed towards the targeted vascular branch for a maximum of 10 min. The device was then transferred back for a full scan to determine the percentage of beads in each region of interest (ROI).

### Simulation of swarms in arbitrary models

From the rolling swarm radius distribution (Fig. 3b), the mass of each µwheel was estimated assuming hexagonally close packed beads and the distribution normalized by the total swarm mass. The geometry of the model was characterized with ${\upphi }_{\mathrm{i}}\left({x}_{i}\right)$ using the position and incline angle at each junction. Using linear interpolation between junction points, the angle at any position in the network could be determined. For the case where a turn was followed by another turn, a separate incline function was defined for each branch. For each simulation, the swarm was initialized at the start position with each µwheel at $x=0$ and then numerically integrated using Euler’s method with a time step of 0.1 s and Supplemental Equation 1 using ${\upphi }_{\mathrm{i}}\left({x}_{i}\right)$ at the µwheel current position. After each step, the mass weighted position mean of the entire swarm was used to identify the swarm centroid location. This process was continued until the centroid position was greater than the desired turn location. µWheels that successfully continued targeting were those within 0.5 $\upsigma$ of the turn point, where $\upsigma$ is the weighted standard deviation of the swarm. For the case where there was a parallel channel nearby, the µwheels within 0.5 $\upsigma$ of the parallel channel were considered lost (Fig. 5b). This procedure was continued until the swarm made all necessary turns to reach the target branch. To simulate collection in a target branch, the swarm was integrated forward until the mass weighted mean advanced 1 mm where all mass remaining was counted as successfully targeted.

### Bead quantification and targeting efficiency

For all fluorescent quantification, each 16-bit image was first thresholded to remove the background noise (pixels with intensity < 3600) thereby isolating the bead fluorescent signal. To determine the fluorescence per bead, a glass slide with fluorescently labeled beads was placed on the same microscope in which scans of the device were performed. A picture was taken with the same settings and light source intensity. The beads were then manually counted and divided by the total brightness signal in the image to calculate the number of beads per fluorescent count. For targeting experiments, ROI masks for each branch were created and exported using Fiji^50^. Using Python, the targeting efficiency was calculated by dividing the fluorescence intensity in the target ROI after targeting by the total fluorescence intensity before targeting.

## Supplementary Information

Supplementary Information 1.

Supplementary Video 1.

Supplementary Video 2.

Supplementary Video 3.

Supplementary Video 4.
