# Supplementary material for: Multimodal microwheel swarms for targeting in three-dimensional networks
Source: Sci Rep. 2022 Mar 24;12:5078. doi: 10.1038/s41598-022-09177-x (PMC8948265; doi:10.1038/s41598-022-09177-x)
Supplement: Supplementary file 1 — Supplementary Information 1. [file 41598_2022_9177_MOESM1_ESM.pdf]

Multimodal Microwheel Swarms for Targeting in Three-Dimensional Networks

C.J. Zimmermann,[1] Paco S. Herson[2], K.B. Neeves[3,4], D.W.M. Marr[1,*]

[1]Department of Chemical and Biological Engineering, Colorado School of Mines

[2]Department of Anesthesiology, University of Colorado Denver | Anschutz Medical Campus

[3]Department of Bioengineering, University of Colorado Denver | Anschutz Medical Campus

[4]Department of Pediatrics, University of Colorado Denver | Anschutz Medical Campus

[*]Corresponding author

Supplemental Information

Expanded Velocity Expression

$$V(R,\phi) = \frac{R(R^3 mg\delta \sin(\phi) - 16Rmg\delta(R+\delta)^2 G\sin(\phi) + a^3\gamma\eta(-R^2 + 16(R+\delta)^2 G)}{a^3\eta(-R^3 + 16R(R+\delta)^2 G + 128\pi\delta k_2 (R+\delta)^2)} \quad (SE1)$$

where

$$G = \ln\left(\frac{R+\delta}{R} + \ln(2)\right) \quad (SE2)$$

Magnetic Field Equations

For all experiments, the circular constant magnitude rotating magnetic field was generated using

$$\omega = 2\pi f$$

$$\mathrm{V}_x = \mathrm{V}_0[\cos(h)\cos(\omega t) - \sin(\theta)\sin(h)\sin(\omega t)]$$

$$\mathrm{V}_y = -\mathrm{V}_0[\sin(h)\cos(\omega t) + \sin(\theta)\cos(h)\sin(\omega t)]$$

$$V_z = C * V_0 \cos(\theta) \sin(\omega t) \qquad (SE3)$$

where V_x, V_y and V_z are voltages applied to each set of coils, V_0 is the voltage multiplier, h the heading angle, θ the camber angle, f the field frequency, t time, and C a coefficient to correct for a different coil distance between the Z coils and the sample compared to the X-Y coils.

Swarm Actuation Patterns

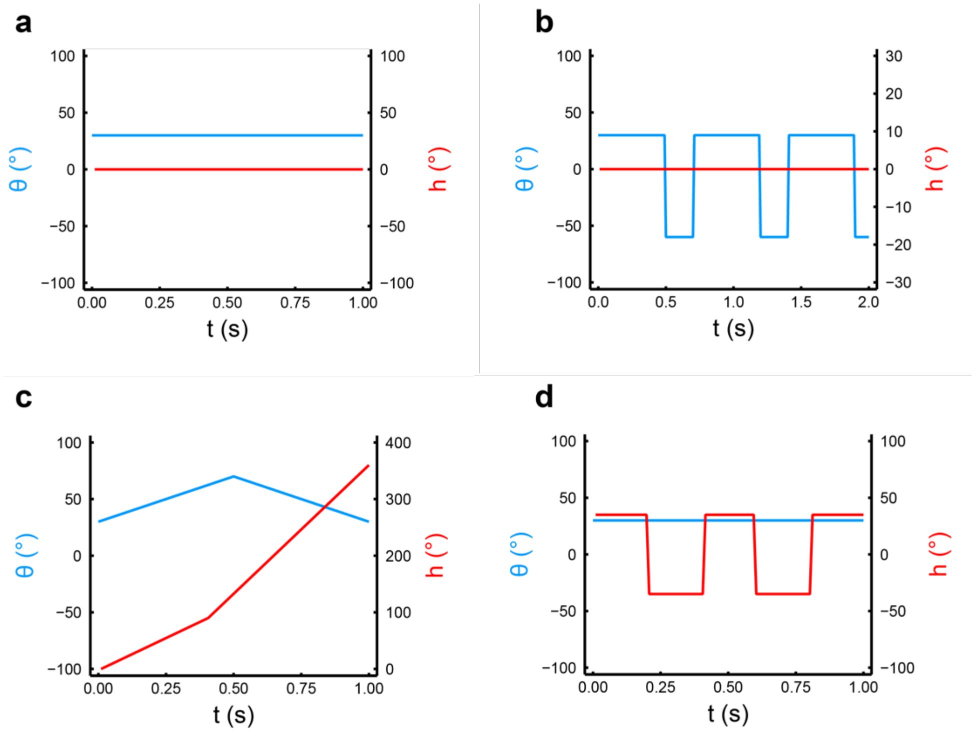

Fig S1. Actuation patterns for swarm modes. The circular field is modified by changing its heading angle h and camber angle θ in time to create the swarm fields. a) Rolling b) Flipping c) Corkscrew d) Switchback.

Separation Widths

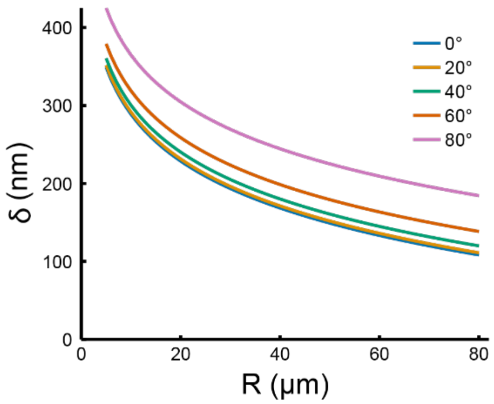

Fig S2. Calculated μwheel separation widths.

3D-Printed Model Roughness

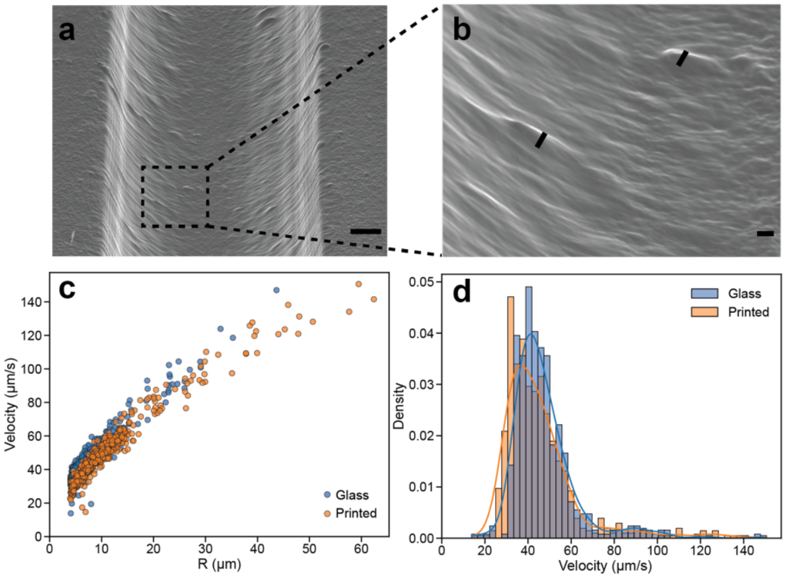

Fig S3: μWheel travel on 3D printed surfaces a) Scanning electron microscopy of 3D-printed half cylindrical channel. Scale = 100 μm. b) Increased magnification of (a). Scale = 10 μm. c) μWheel velocity on glass and flat 3D-printed surfaces (field = 3.7 mT, 40 Hz, and $\theta = 30°$). d) μWheel velocity probability density distribution.
